# Recent Advances in the Study of Gas Vesicle Proteins and Application of Gas Vesicles in Biomedical Research

**DOI:** 10.3390/life12091455

**Published:** 2022-09-19

**Authors:** Felicitas Pfeifer

**Affiliations:** Microbiology and Archaea, Department of Biology, Technical University Darmstadt, Schnittspahnstrasse 10, 64287 Darmstadt, Germany; pfeifer@bio.tu-darmstadt.de; Tel.: +49-6151-1623670

**Keywords:** gas vesicles, *Halobacterium salinarum*, protein nanostructures, acoustic biosensors, vaccine development

## Abstract

The formation of gas vesicles has been investigated in bacteria and haloarchaea for more than 50 years. These air-filled nanostructures allow cells to stay at a certain height optimal for growth in their watery environment. Several *gvp* genes are involved and have been studied in *Halobacterium salinarum,* cyanobacteria, *Bacillus megaterium*, and *Serratia* sp. ATCC39006 in more detail. GvpA and GvpC form the gas vesicle shell, and additional Gvp are required as minor structural proteins, chaperones, an ATP-hydrolyzing enzyme, or as gene regulators. We analyzed the Gvp proteins of *Hbt. salinarum* with respect to their protein–protein interactions, and developed a model for the formation of these nanostructures. Gas vesicles are also used in biomedical research. Since they scatter waves and produce ultrasound contrast, they could serve as novel contrast agent for ultrasound or magnetic resonance imaging. Additionally, gas vesicles were engineered as acoustic biosensors to determine enzyme activities in cells. These applications are based on modifications of the surface protein GvpC that alter the mechanical properties of the gas vesicles. In addition, gas vesicles have been decorated with GvpC proteins fused to peptides of bacterial or viral pathogens and are used as tools for vaccine development.

## 1. Introduction

Several bacteria and some euryarchaeota produce gas vesicles, protein nanostructures filled with air. Gas vesicles are spindle- or cylinder-shaped, with a width of 100–250 nm and a length of up to 2 µm. Gas molecules dissolved in the cytoplasm freely permeate in and out via small holes, and the hydrophobic gas-facing interior surface ensures that water molecules are repelled [1]. The shell is only formed by proteins; lipids and carbohydrates are absent. Cells might contain a few or more than a hundred gas vesicles. They scatter light and allow cells to counteract sinking and staying at positions optimal for light or nutrient supply [2]. Approximately 3–10% of the cell volume must be occupied by gas vesicles to provide buoyancy [3,4]. Both gas-vesiculated (Vac^+^ phenotype) and non-gas-vesiculated cells are similar in size, but the large surface area in relation to the cytoplasmic volume enhances the uptake of nutrients in cells tightly filled with gas vesicles.

Gas vesicles produced by photosynthetic cyanobacteria such as *Anabaena flos-aquae, Planktothrix* species or *Microcystis aeruginosa* allow the cells to float and stay near the surface to convert light into chemical energy. Spherical colonies of *Microcystis* with diameters of 2–4 mm exhibit high floating velocities of 1 mm per second, and the sinking velocity allows a migration of 8.6 m in 4 h [5]. Heterotrophic bacteria produce gas vesicles, especially when living in cold marine or freshwater habitats [6,7,8]. Even soil bacteria form gas vesicles, such as *Bacillus megaterium* or *Streptomyces*, and also the enterobacterium *Serratia* sp. ATCC39006 isolated from brackish water [9,10,11]. *Serratia* uses gas vesicles and flagella to move, and both features are oppositely regulated so that a simultaneous synthesis of flagella and gas vesicles is excluded [11,12]. The inverse regulation is contrary to the presence of gas vesicles and archaella (the archaeal flagella) in the extremely halophilic archaeon (haloarchaeon) *Halobacterium salinarum*, where Vac^+^ cells swim and float. Besides *Hbt. salinarum*, the moderately halophilic *Haloferax mediterranei* and the square *Haloquadratum walsbyi* contain gas vesicles [13,14,15,16]. Haloarchaea thrive in the Dead Sea, salt lakes, or salterns and adapt to the molar sodium chloride concentrations by balancing the internal potassium chloride concentrations by the “salt-in” strategy. The cytoplasm contains up to 5 M salt, mainly as potassium chloride ions. *Halobacterium* species use the light-driven proton pump bacteriorhodopsin to convert light into chemical energy. Since gas vesicles scatter light waves, they protect the cells against the damaging UV light. The haloarchaeal gas vesicle formation is enhanced in the presence of low light intensity, high-salt concentrations (>17% salt), and low temperature (15 °C) [17,18]. Only a few and smaller gas vesicles occur in *Hbt. salinarum* cells grown anaerobically by arginine fermentation; the low energy supply under these conditions presumably causes the reduced production [19,20]. Additionally, methanogenic archaea such as *Methanosarcina barkeri* FR-1 and *M. vacuolata* produce gas vesicles. Gas vesicles of *M. barkeri* are only observed in cells grown with H_2_-CO_2_, suggesting that the gas vesicle protein (*gvp*) genes might be expressed in response to hydrogen gradients [21]. Other *Methanosarcina* strains contain gas vesicles when grown with methanol and acetate.

The *gvp* genes are always arranged in gene clusters and have been studied in several species (Table 1). Haloarchaea contain the fourteen *gvp* genes as oppositely arranged *gvpACNO* and *gvpDEFGHIJKLM* clusters in the vac region. *Hbt. salinarum* PHH1 harbors two related vac regions on plasmid pHH1 (p-vac) and a mini-chromosome (c-vac) [22], whereas *Hbt. salinarum* NRC-1 contains two copies of the *gvp1* gene cluster (related to p-vac) and a single copy of *gvp2* (related to c-vac) on two mini-chromosomes [23]. The 8-kDa GvpA encoded by *gvpACNO* forms the major constituent of the gas vesicle shell. GvpA aggregates in a highly ordered arrangement and forms a helix of low pitch, seen as ribs running perpendicular to the long axis by electron microscopy [2,24]. The larger, hydrophilic GvpC protein is attached to the exterior surface and provides reinforcement for the shell [2,25]. The additional Gvp proteins are minor constituents of the shell, serve as chaperones, or deliver energy, such as the AAA-ATPase GvpN (Table 2). Two regulator proteins are involved: GvpE activates the transcription, and GvpD has a repressing function [25]. GvpD, GvpE, GvpO, and GvpI are haloarchaea-specific proteins. The *gvp* gene clusters of the methanogenic archaea contain three to four identical copies of *gvpA*, and the arrangement of the *gvp* genes is less dense. The *gvp* gene clusters lack *gvpDE* but also *gvpC* and *gvpI* and resemble in these features more the cyanobacterial *gvp* gene clusters (Table 1). It is possible that the *gvp* gene cluster has been acquired from other strains, since the *gvp* gene clusters of *Hbt. salinarum* (p-vac) and *M. barkeri* are flanked by insertion sequences or transposons [20,21].

In bacteria, the *gvp* gene cluster of *Anabaena flos-aquae* contains seven identical copies of *gvpA*, together with eight additional *gvp* genes (Table 1) [26,27]. *Bacillus megaterium* harbors the *gvpAPQBRNFGLSKJTU* gene cluster [9] and *Serratia* contains two gene clusters, including three genes encoding specific regulatory proteins: *gvrA, gvrB*, and *gvrC* (Table 1) [11,28]. GvpV, W, X, Y, and Z are bacteria-specific proteins and not essential for gas vesicle formation. The *gvp* genes conserved in all *gvp* gene clusters are *gvpA*, *gvpC,* and *gvpFGKLN.* The latter gene encodes the AAA-ATPase GvpN (Table 2). GvpF and GvpL are related proteins playing an important role in protein complex formation (see below). Additionally, two genes related to *gvpA* are present but often named differently; the proteins encoded are GvpJ and GvpM in haloarchaea and GvpB and GvpS (related to GvpM) in *Bacillus*, as well as GvpA_2_ (M) and GvpA_3_ (J) in *Serratia*. Balanced amounts of the different accessory Gvp are important; an overexpression of single *gvp* genes in addition to the entire *gvp* gene cluster can result in a reduction in the amount and/or the size of gas vesicles, as observed with high amounts of GvpG, H, and M in *Haloferax volcanii* p-vac + X transformants or high amounts of GvpF_1_, F_2_, and A_3_ in the case of *Serratia* [29,30]. The haloarchaeal gas vesicles formation and the different Gvp proteins have been studied in *Hfx volcanii* transformants; the species offers a clean genetic background, since it lacks any *gvp* gene cluster. The original strain *Hbt. salinarum* PHH1 harbors two related *gvp* gene clusters that are differently expressed, grows much slower than *Hfx. volcanii* (generation time: 8 h instead of 3 h), and is more difficult to transform.

This review summarizes recent results on gas vesicle formation and the interaction potential of the different haloarchaeal gas vesicle proteins. Additionally, the development of recombinant gas vesicles for applications in biomedical research will be highlighted.

## 2. Gas Vesicle Proteins and Genes of *Hbt. salinarum*

**Figure 1:**
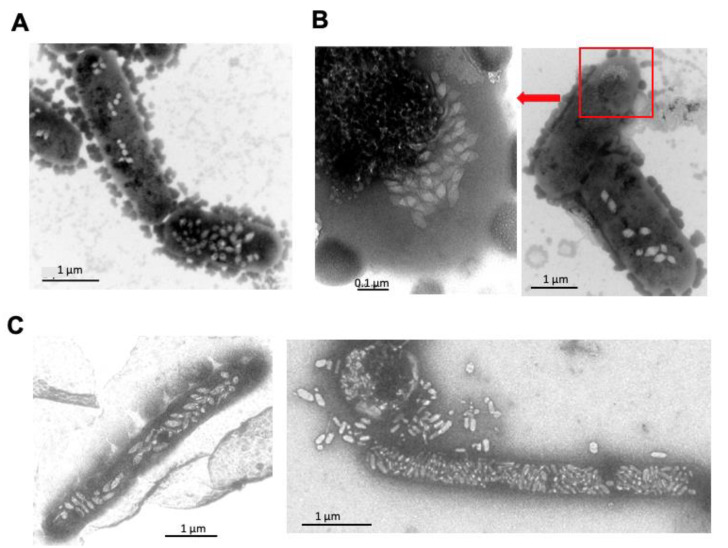
Inspection of *Hbt. salinarum* cells containing gas vesicles by transmission electron microscopy. Gas vesicles are seen as white bodies inside the cells. (**A**,**B**) Younger cells derived from a liquid culture grown to an optical density of 0.78. The red box in (**B**) places the enlarged image at the left side in the image on the right. (**C**) Old cells derived from a surface layer of a liquid culture left standing on the bench for five months (Faist and Pfeifer, TU Darmstadt).

The extremely halophilic archaeon *Hbt. salinarum* produces spindle-shaped gas vesicles by expression of the *gvp* genes of the p-vac region. Younger cells contain a few gas vesicles in small clusters or harbor >30 gas vesicles per cell (Figure 1A). This observation could reflect a difference in age of the cells in the batch culture; the younger cells are not yet filled with gas vesicles. In some cells, a cluster of many small, spindle-shaped structures is observed (Figure 1B), raising the question of whether these particles are all synthesized at once, grow, and are distributed in the cell. Old cells, obtained from a surface layer of a culture standing for five months on the bench, are filled with many large gas vesicles that reduce the cytoplasmic volume of the cells (Figure 1C). Due to the high-salt concentration of 5 M in the cytoplasm, the cells easily lyse in water, and gas vesicles are released. They are rigid and stable and do not collapse when inspected by electron microscopy.

The fourteen genes required for gas vesicle formation are arranged in two oppositely oriented gene clusters, *gvpACNO* and *gvpDEFGHIJKL*. A low expression of *gvpACNO* and *gvpFGHIJKLM* in the exponential growth phase results in a few gas vesicles [39]. The *gvpFGHIJKLM* genes are transcribed during exponential growth in minor amounts only. The weak *P_pF_* promoter is located in the middle of the *gvpE* reading frame and not induced by the activator protein GvpE [39,40]. The accessory proteins GvpF through GvpM are thus produced in minor amounts only. In the stationary growth phase, large amounts GvpA and GvpC occur due to a 10-fold activation of the *P_pA_* promoter by GvpE [40]. The promoter activities of the p-vac region were monitored earlier using the halophilic β-galactosidase BgaH as the reporter [37,38,41,42]. Recently, we quantified these promoter activities in living cells using a salt-adapted GFP as the reporter and studied the activities in relation to the strong ferredoxin promoter, *P_fdx_*, of *Hbt. salinarum* [40]. The basal activities are low; the promoter of *gvpACN*, *P_pA_*, reaches only 1/10 of the *P_fdx_* activity, but the activation by GvpE leads to a 10-fold enhancement. In addition to *P_pA_*, GvpE activates *P_pD_* and, thus, autoregulates the expression of *gvpDE*. *P_pD_* exhibits a much lower basal activity, i.e., only 1/60 of *P_pA_* during stationary growth, but is also 10-fold induced by GvpE. The *gvpA* transcript is produced in much larger amounts compared to the *gvpACN* co-transcript, and the translation leads to large amounts of GvpA constituting the gas vesicle shell. The *gvpN* mRNA is present in lower amounts (due to a termination signal between *gvpA* and *gvpC*). GvpN is a AAA-ATPase and able to hydrolyze ATP, as determined for the cyanobacterium *Anabaena* sp. PCC 7120 [32]. This protein is required for the enlargement of gas vesicles, since *Hfx. volcanii* ∆N transformants (carrying except for *gvpN* all of the remaining *gvp* genes of p-vac) produce tiny gas vesicles only [31]. The transcription of *gvpO* starts at a separate promoter, *P_pO_*, that is active throughout the growth, but the function of GvpO is not yet known. The protein is essential, since ∆O transformants are Vac^−^.

Gas vesicles are easy to isolate by lysis of the cells in water and centrifugation-enhanced flotation, and they are stable for many months when stored in buffer solutions in the refrigerator. Their protein content cannot be determined by SDS-polyacrylamide gel electrophoresis (SDS-PAGE), since gas vesicles do not disintegrate in sodium dodecyl sulfate-containing aqueous solutions. Only GvpC is released from the outer surface of cyanobacterial or haloarchaeal gas vesicles [43,44,45,46,47,48]. The hydrostatic pressure required to collapse cyanobacterial gas vesicles is three-fold decreased when GvpC is rinsed off with 6 M urea. In haloarchaea, GvpC is released by rinsing gas vesicles with water [46]. However, Western analyses with lysates of *Hfx. volcanii* transformants producing single Gvp proteins visualize monomers of any Gvp protein by SDS-PAGE [14,29,46,49,50]. Even the hydrophobic GvpA and the related GvpJ and GvpM are detected as monomers, and oligomers of GvpJ and GvpM smear the top, whereas many aggregates of GvpA appear near the top of the gel. The expression vector pJAS35 is used in these studies, where the *gvp* reading frame is expressed under the control of the strong *P_fdx_* promoter [51]. His-tagged Gvp proteins (_His_X) synthesized in *Hfx. volcanii* under native conditions can be isolated by affinity chromatography on Ni-NTA agarose matrices, but some of the Gvp proteins precipitate during the isolation procedure and need to be refolded in high-salt solutions [29,52]. In vitro protein–protein interaction studies are thus not easy to perform with these proteins.

## 3. Interactions of Gvp Proteins Studied by Split-GFP and Pulldown Assays

Due to these problems, we investigated protein–protein interactions in vivo in *Hfx. volcanii* transformants, where the high-salt concentrations (3–5 M KCl) of the cytoplasm offer native conditions [52]. Two different methods have been applied: (i) a split-GFP analysis, where the protein–protein interaction is measured and quantified by the assembly of a fluorescent GFP, and (ii) pulldown assays with a bait protein tagged with the cellulose binding domain (CBD) of *Clostridium thermocellum* and a selection of the putative binding partners in the cytoplasm of the respective transformants with a cellulose matrix. Both methods are adapted to the high-salt concentrations of the haloarchaeal cell.

The split-GFP analysis has been developed to investigate protein–protein interactions in bacteria and yeast [53,54,55]. The green fluorescent protein GFP is split into two fragments: NGFP containing the fluorophore and CGFP. Cells producing the two fragments in trans are not fluorescent, and both fragments only assemble when fused to interacting proteins. For the split-GFP analysis in haloarchaea, the salt-adapted mGFP2 exhibiting a higher fluorescence compared to smGFP [40,56] was split into the two fragments NGFP and CGFP and fused to the two putative Gvp interaction partners under investigation [52]. Only when the two Gvp proteins interact, the fragments will assemble a fluorescent GFP protein, and the fluorescence is quantified using a phosphorimager. The method is limited to soluble proteins; hydrophobic proteins such as GvpA, GvpJ, or GvpM (A-J-M family) have the tendency to form inclusion bodies when produced without chaperones in the cells. GvpM fused to the entire GFP forms aggregates in *Hfx. volcanii* transformants [57], and such aggregates interfere with the assembly of the mGFP2 fragments and result in a low relative fluorescence (rf value) of the respective transformants (Table 3). Rf values < 5 are regarded as weak interactions, and transformants harboring GvpA, GvpJ, or GvpM yield even smaller rf-values < 1. Due to these limitations, the interaction of GvpA molecules in the gas vesicle shell cannot be investigated using split-GFP; even the dimerization of fragments consisting of α-helix 1 or 2 of GvpA that include polar amino acids (aa) are not observable [58]. A real exception is the interaction of GvpA with GvpF determined in A/F transformants (rf 20) (Table 3) [50]. The 23-kDa GvpF appears to bind monomeric GvpA. GvpF either acts as a chaperone to keep GvpA soluble and/or enables the oligomerization of GvpA molecules during the formation of the ribbed shell.

Similarly, the GvpA-related GvpJ and GvpM both interact with GvpL, a protein related to GvpF (J/L, rf 11; M/L, and rf 12), and other interaction partners are not observed (Table 3) [50]. In contrast, many interactions are found with the soluble 32-kDa GvpL (C,N,O and F,G,H,I,J,K,M), and the AAA-ATPase GvpN (C,N,O and F,G,H,I,J,K,L,M); both proteins contact any Gvp involved in the formation of the gas vesicle shell, except for GvpA (Table 3). Additionally, the surface-attached GvpC protein (C,N,O and F,H,I,K,L) and GvpF (A,C,O and G,H,I,L) have several interaction partners. The least contacts are found with GvpG (L,F) and GvpK (C,L) [50,58]. These data lead to the hypothesis that all Gvp proteins might form (a) complex(es) in early stages of gas vesicle assembly. The complex might initiate the formation of the gas vesicle caps starting at the tips, and/or ensure that the caps are enlarged until the final width of 200 nm is reached.

The pulldown assays applied as second method confirm these interactions and suggest additional contacts of the hydrophobic A-J-M Gvp proteins [50,58]. For the pulldown experiments, one of the Gvp proteins is fused to CBD at the N- or C-terminus (_CBD_X or X_CBD_) and used as bait to select the putative interaction partner in the respective *Hfx. volcanii*_CBD_X + Y transformants (X, Y = GvpA through GvpO). The bait and prey proteins are selected and purified by binding to a cellulose matrix via CBD. Using this approach, GvpA, GvpJ, and GvpM interact with Gvp F, G, K, and L and the heterodimer H-I but also with each other (A-J, A-M, and J-M) (Table 4), whereas, in the split-GFP analysis, only the A/F, or J/L and M/L, interactions are observed. A putative complex formation was accessed using _CBD_M or _CBD_A as bait and synthesized together with all other Gvp proteins in the same cells. All Gvp proteins are selected by both baits, although GvpI, K, or L do not directly bind GvpA. The results imply that any of the Gvp proteins contact GvpA (or GvpM) either directly or via another binding partner [50,58]. It is likely that the proteins form complex(es) during the formation of gas vesicles.

Surprisingly, GvpA binds GvpN and GvpO, but we cannot identify any interaction between GvpA and GvpC, although GvpC attaches to the exterior surface of the shell formed by GvpA [58]. Neither the combination _CBD_A-C or _CBD_C-A yields an interaction of GvpA and GvpC. One reason might be that the interaction site of GvpC in GvpA is hidden by the large CBD moiety fused to the N-terminus of the 8-kDa GvpA or that GvpC requires the ordered arrangement of GvpA in the gas vesicle shell to interact. In the case of the _CBD_C-A experiment, the nonspecific aggregation of GvpA might prevent an interaction with GvpC, but the monomers and aggregates of GvpA are selected by GvpN and GvpO in _CBD_N-A and _CBD_O-A transformants, demonstrating contacts of both proteins to GvpA [58]. It might be interesting to inspect the complexes formed by _CBD_N-A and _CBD_O-A in comparison to _CBD_C-A by electron microscopy.

## 4. Effect of Mutations in GvpA, GvpJ, and GvpM

**Figure 2:**
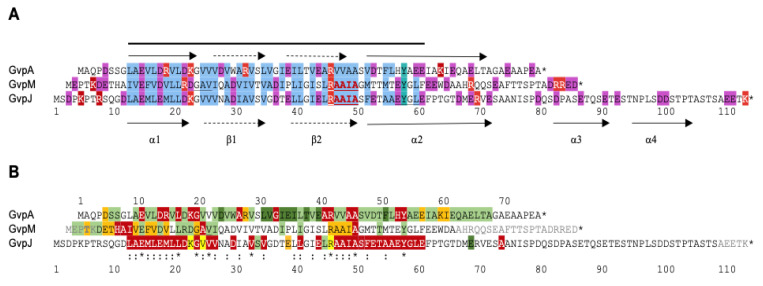
Comparison of GvpA, GvpJ, and GvpM of *Hbt. salinarum*. (**A**) Alignment of the three aa sequences highlighting the conserved regions (marked by a bar on top). Polar aa are indicated in red (K,R) or purple (D,E) and nonpolar aa in blue. The putative secondary structure is marked by arrows (α-helices α1–α4, and β-sheets β1 and β2). (**B**) Comparison of the results on the different ∆X + X_mut_ transformants (X = A, J, or M) with respect to the Vac phenotype. Vac^+^ transformants are shaded in green (spindle-shaped wild type gas vesicles in light green, cylinder-shaped gas vesicles in dark green), Vac^±^ transformants in orange, and Vac^−^ transformants in red. Amino acid substitutions in GvpJ, leading to unstable gas vesicles, are marked in yellow. Residues not shaded are not explored. (*) and (:) below the alignment highlight conserved positions (after [61]).

The three proteins GvpA (76 aa), GvpJ (114 aa), and GvpM (84 aa) are members of the A-J-M family and exhibit sequence similarities of 50% (A-J), 48% (A-M), and 60% (J-M) (Figure 2A). GvpA is the major gas vesicle structural protein produced in large amounts, whereas the other two are present in lower amounts during the early stages of gas vesicle assembly only. All three are essential and take part in the initial steps [31]. An in silico structural model of GvpA was obtained [59,60], and 3D structural models of GvpJ and GvpM were obtained by homology modeling [52]. The secondary structure predictions suggest up to four α-helices (α1–α4) and two antiparallel β-sheets separating α1 and α2 (Figure 2A). The β-sheets of GvpA most likely constitute the hydrophobic interior gas-facing surface of the gas vesicle shell. The three proteins differ in length, and 20 aa of the C-terminus of GvpM can be deleted without an effect on the Vac^+^ gas vesicle phenotype. In contrast, only 5 aa of the C-terminus of GvpJ can be deleted; the remaining sequence is essential for the function of GvpJ [61]. The N-terminal portion of GvpJ is conserved between archaea and bacteria and also in relation of GvpA and GvpM, but the aa between positions 81 and 114 are *gvp* cluster-specific (Figure 2B).

The effect of single substitutions on gas vesicle formation was tested for the first 60 aa in *Hfx. volcanii* ∆A + A_mut_, ∆J + J_mut_, or ∆M + M_mut_ transformants [58,61,62]. The ∆X construct (X = A, J, or M) always contains the *gvp* genes of the p-vac region, except for the *gvpA* (∆A)*, gvpJ* (∆J)*,* or *gvpM* (∆M) reading frames. The Vac phenotypes of the transformants obtained are summarized in Figure 2B. Approximately two-thirds of the 60 single aa substitutions tested in GvpA yield Vac^+^ transformants that contain gas vesicles of wild-type shapes (marked in green in Figure 2B); only nine of them harbor cylinder-shaped gas vesicles instead of the normal spindle-shaped structures [62]. A few of the ∆A + A_mut_ transformants contain mini-gas vesicles, and 14 transformants are gas vesicle-negative (Vac^−^) (Figure 2B). It is remarkable that only a single aa substitution in GvpA results in long and cylinder-shaped gas vesicles, tiny gas vesicles, or even in the lack of these structures. However, also, the difference between the spindle-shaped gas vesicles derived from p-vac and the cylinder-shaped gas vesicles of c-vac in *Hbt. salinarum* is due to the three aa alterations observed in GvpA [63]. In the case of GvpM, the aa substitutions either yield Vac^+^ or Vac^−^ ∆M + M_mut_ transformants; changes in the gas vesicle shape are not observed. Only the amount of gas vesicles is reduced in half of the cases (Vac^±^), and five are Vac^−^ [61]. The largest number of Vac^−^ transformants occurs with variants of GvpJ, where 34 out of 41 ∆J + J_mut_ transformants are Vac^−^ (Figure 2B). Three of these transformants contain unstable gas vesicles underlining that GvpJ is part of the gas vesicle shell. The large amount of Vac^−^ transformants suggest that GvpJ is important and participates in several steps in gas vesicle formation.

Despite the high conservation in the N-terminal portion of the three proteins, except for an alanine residue at the end of the conserved RAAIA-motif of GvpJ and GvpM and the related RVVAA motif in GvpA, none of the alterations of the three A-J-M proteins yields a similar Vac phenotype (Figure 2B) [61]. Altering the polar aa in α1 of GvpA yields Vac^−^ transformants, whereas a similar alteration in α1 of GvpM does not affect the Vac phenotype. Similarly, alteration of a nonpolar, hydrophobic aa in α1 of GvpA does not affect the gas vesicle formation in ∆A + A_mut_ transformants, but a similar alteration in α1 of GvpM disturbs the gas vesicle synthesis in ∆M + M_mut_ transformants. In GvpJ, the substitution of any aa in α1 yields Vac^−^ transformants, underlining the importance of this helix for the function of GvpJ [61]. The *gvpJ* gene is found in any gas vesicle gene cluster of archaea or bacteria. However, the divergent C-terminal half of the haloarchaeal GvpJ defines the protein as vac region-specific. Overall, the results on the three A-J-M proteins demonstrate that they have different functions and, despite their relationship, cannot substitute each other.

## 5. GvpC Reinforces the Shell and Shapes the Gas Vesicles

GvpC is the structural gas vesicle protein located at the exterior surface. The protein was first identified in *Anabaena flos-aquae* in A. Walsby’s lab [64]. The 193-aa sequence of *Anabaena* GvpC contains five highly conserved 33-aa repeats that might interact with the periodic structure provided by the aggregated GvpA at the surface of the shell. When GvpC is removed by rinsing the gas vesicles, the critical collapse pressure decreases from 0.55 to 0.19 MPa and raises again to 0.53 MPa when stripped gas vesicles are reconstituted with recombinant GvpC, indicating that GvpC increases the strength and stabilizes the gas vesicle structure [44]. Antibodies raised against GvpC label both the conical end caps and the central cylinders of the native *Anabaena* gas vesicles, and the molar ratio has been found to be 25 GvpA to 1 GvpC [65]. Deletions in GvpC resulting in versions containing only the first two, three, or four of the 33-aa repeats all bind and reinforce the gas vesicles. However, a GvpC protein containing only two repeats binds at a lower ratio and restores less of the strength [48]. Thus, the number of repeats in GvpC influences the strength but, also, the shape of the gas vesicles, as determined for *Microcyclus aeruginosa*. In this case, the number of the 33-aa repeats in GvpC correlates with the diameter of the gas vesicle and inversely with its strength [66]. Wider gas vesicles have a lower critical hydrostatic collapse pressure. Dunton et al. [67] also studied how GvpC binds to the surface of the shell. After incubating *Anabaena* gas vesicles with trypsin and investigating the tryptic peptides by MALDI-TOF MS, GvpA is only cleaved at sites near the N-terminus (within helix α1 that is thus accessible from the exterior side), whereas GvpC is cleaved at most of its potential tryptic sites. Many GvpC peptides remain attached to the GvpA shell, especially both ends of the 33-aa repeats of GvpC that function as the binding site [67]. Taking these results into account, it is surprising that we cannot demonstrate an interaction of the haloarchaeal GvpA and GvpC by split-GFP or pulldown assays [58].

**Figure 3:**
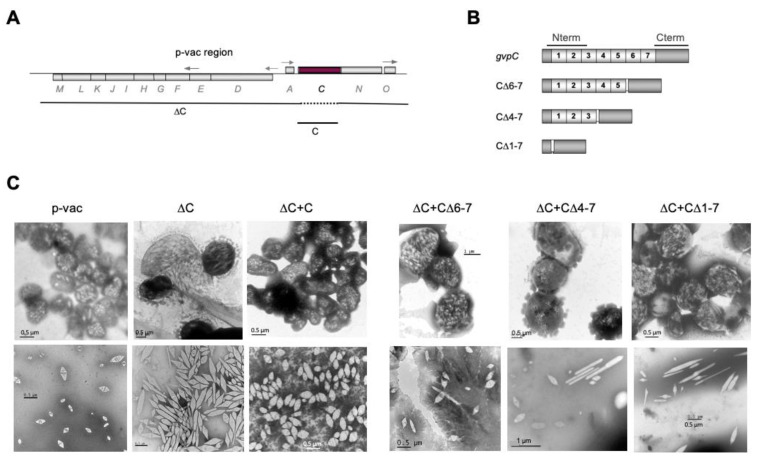
Gas vesicles obtained in *Hfx. volcanii* ∆C and ∆C + C_mut_ transformants. (**A**) Genetic map of the p-vac region derived from *Hbt. salinarum*. Arrows indicate the direction of the transcription. The ∆C lacks the *gvpC* reading frame, and the C construct contains *gvpC* inserted in pJAS35 for the expression. (**B**) Different versions of GvpC lacking 3, 4, or 7 of the aa repeats labeled 1–7. The Nterm and Cterm fragments of GvpC used for protein–protein interaction studies are marked on top. (**C**) Transmission electron micrographs of *Hfx. volcanii* cells (upper lane) and isolated gas vesicles (lower lane) derived from the respective transformants (Faist and Pfeifer, TU Darmstadt).

**Figure 4:**
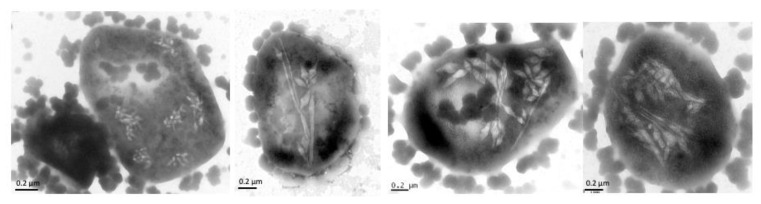
*Hfx. volcanii* ∆C + C∆4–7 transformants inspected by transmission electron microscopy after 32 d of growth on solid media (Faist and Pfeifer, TU Darmstadt).

For *Hbt. salinarum*, the function of GvpC was investigated in ∆C transformants containing a construct harboring the p-vac region that incurred a deletion of the *gvpC* reading frame (Figure 3A) [24]. *Hfx. volcanii* ∆C transformants produce large amounts of irregularly shaped gas vesicles up to 1.6 µm in length, and the complementation of ∆C with GvpC results in spindle-shaped gas vesicles similar to the wild type in ∆C + C transformants (Figure 3C). The 42.3-kDa GvpC of *Hbt. salinarum* is larger than the 21.9-kDa *Anabaena* GvpC and contains seven less conserved repeats of 32–39 aa. Deletions of two, four, or all of these repeats result in GvpC variants C∆6–7 (contains repeats 1–5), C∆4–7 (repeats 1–3), and C∆1–7 consisting of the globular C-terminal domain only (Figure 3B). Using these GvpC variants to complement ∆C in *Hfx. volcanii* transformants, gas vesicles of altered shapes are observed (Figure 3C). The ∆C + C∆1–7 and ∆C + C∆6–7 transformants are tightly filled with gas vesicles, whereas ∆C + C∆4–7 transformants produce odd-shaped gas vesicles with rosette-like structures (Figure 3C). The spindle-shaped portion of these gas vesicles is often attached to a long, thin structure (25–50 nm in width and up to 1.2 µm in length) (Figure 4). Since the cells are not tightly filled with gas vesicles, these extended structures are not formed because of space limitations. It is possible that the two remaining repeats in C∆4–7 allow only the formation of gas vesicles with these small diameters. The shape of the gas vesicles in ∆C + C∆6–7 transformants (two repeats are missing) mostly resembles that of the wild type, whereas the gas vesicles of the ∆C + C∆1–7 transformants, formed in the presence of the globular C-terminal portion of GvpC, are long and thin (on average, 880 nm × 140 nm), and differ from the gas vesicles of the ∆C transformants (680 × 180 nm) formed without GvpC (Figure 3B) [24]. Presumably, the globular C-terminal portion of GvpC present in the ∆C + C∆1–7 transformants still binds to the shell and causes the reduction of the gas vesicle width, similar to the results on *Microcystis aeruginosa*, where the number of repeats correlate with the diameter of the gas vesicle [66].

Interaction studies with the haloarchaeal GvpC by split-GFP yield that the protein binds many other Gvp (F,H,I,K,L,N,O) (Table 3) [58]. Fragments of GvpC harboring the first three helical repeats (Nterm, aa 10–130) or the global C-terminal portion (Cterm, aa 329–388) (Figure 3B) were used to confine these interactions. Fragment Nterm bound GvpC, L, and H, whereas Cterm bound GvpC, F, H, I, L, N, and O [58]. Additionally, we investigated whether GvpC is able to dimerize. The interactions Cterm/Cterm and Cterm/Nterm suggest the dimerization of GvpC via these portions, but Nterm/Nterm interactions are not detectable [58]. Thus, GvpC molecules might form a mesh at the surface of the gas vesicle. Additionally, the interaction of the two GvpC fragments was tested with fragments of GvpA, and Nterm yields the highest rf value (rf 3.5), with A1–22 containing the α1 of GvpA, whereas all other GvpA fragments resulted in lower rf values. The α-helical repeats of GvpC present in Nterm might bind α1 of GvpA, similar to the results obtained for the cyanobacterial GvpC fragments bound to gas vesicles after tryptic digestion [67]. Interestingly, the presence of GvpC, GvpN, and GvpO enhances the dimerization of GvpA when analyzed by split-GFP, suggesting that the proteins suppress an unspecific aggregation of GvpA and support the formation of the gas vesicle shell [58].

## 6. Implications for Gas Vesicle Assembly

**Figure 5:**
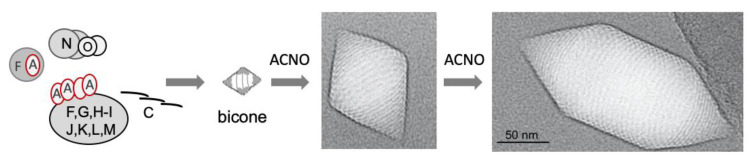
Model for the assembly of gas vesicles. The interacting GvpF–GvpA (F–A) and the complex formation of FGHIJKLM, as well as N–O, are shown. GvpC is presented as a rod able to form dimers or multimers by Nterm/Cterm and Cterm/Cterm interactions. The first gas-filled structure seen in cells by transmission electron microscopy is a small bicone that is enlarged to a spindle-shaped structure. The further addition of GvpA and GvpC yields the cylinder-shaped nanostructure; the formation of the cylinder shape depends on the presence of GvpC. The cryo-electron microscopy of *Hbt. salinarum* gas vesicles was performed by Daniel Bollschweiler and Harald Engelhardt, Max-Planck-Institute for Biochemistry, Martinsried, Germany.

Gas vesicle formation in *Hbt. salinarum* PHH1 starts with the transcription of *gvpFGHIJKLM* and *gvpACNO* in the early exponential growth phase. Some or all of the GvpF through GvpM proteins form a nucleation complex and attract GvpA. Monomeric GvpA is bound by GvpF and GvpO, presumably already before GvpA is incorporated into the shell [50,58]. GvpF and GvpO both interact with GvpL, the major platform binding all Gvp except for GvpA. However, it is not known whether GvpL binds all these Gvp at once or sequentially. GvpL also attracts GvpC by binding at both ends, ensuring that GvpC is present in the complex and can attach to the exterior surface as soon as it forms. The AAA-ATPase GvpN could power the GvpA incorporation or support the subunit-turnover in the complex. The assembly of a gas vesicle presumably starts at the tips of the conical cap structures, and the ribs forming this structure contain aggregated GvpA. The width of the caps increases up to 200 nm and then stays constant in the cylinder portion of the gas vesicles. A model for the assembly is presented in Figure 5.

GvpF has been detected at the gas-facing surface of the cyanobacterial gas vesicle [68], and we could demonstrate that the two residues R28 and E40 located in the β-sheet portion of the haloarchaeal GvpA are important for the A/F interaction [50,62]. Solid-state NMR studies propose that these two amino acid residues also contribute to the A/A interaction in the ribs [69,70]. Thus, R28 and E40 of GvpA might form ion bonds in the A/F interaction prior to the incorporation or support the A/A polymerization in the shell, depending on the presence or absence of GvpF. The presence of a nucleation complex at the tip of the caps also prevents interactions of the hydrophobic gas-facing surface of the shell and might help to initiate the rib formation of GvpA and also close the hole at the tip [58]. The attachment of GvpC to this protein complex ensures that GvpC covers the surface of the gas vesicles from the very beginning [65]. GvpC is a long, rod-shaped protein, and the α-helical aa repeats are supposed to span several GvpA molecules, as well as adjacent ribs, to stabilize the shell [2]. However, a mesh of GvpC could also form by the interaction of the Cterm/Cterm and Cterm/Nterm portions of GvpC. The binding of GvpN and/or GvpO at the Cterm portion of GvpC perhaps interrupts this mesh at the site where GvpA molecules are incorporated to enlarge the shell [58]. Both GvpN and GvpO bind GvpA as well. A crystal structure of GvpC would shed more light on the proposed interactions and the formation of the GvpC multimers. Gas vesicles increase in size up to their stationary growth; since only the *gvpACNO* genes are still expressed, the GvpA, C, N, and O proteins are sufficient to enlarge the already existing gas vesicles.

## 7. Application of Engineered Gas Vesicles

Gas vesicles have gained a lot of interest in biomedical research. They are used to present polypeptides from pathogens on the surface for antigen generation but are also applied as stable contrast agents for ultrasound imaging or used as acoustic biosensors. The mechanical and biochemical properties of gas vesicles can be modulated by GvpC, the protein bound to the gas vesicle surface. GvpC tolerates modifications introduced at the C-terminus or within one of the helical repeats.

For antigen presentation, bacterial or viral polypeptides have been fused to the C-terminus of the haloarchaeal GvpC to display the fusion proteins on the surface of *Hbt. salinarum* gas vesicles [71]. The modified gas vesicles are easy to isolate by lysis of the cells in water and centrifugally assisted flotation and are nontoxic to mammalian cells. Such decorated gas vesicles prove to be an effective antigen display system for biomedical research and diagnostics and stimulate immune reactions in rabbits or mice without the application of toxic lipopolysaccharide or lipoid A as an adjuvant [72]. A long-lived antibody response was obtained against different peptides of the Simian Immunodeficiency Virus, SIV (Tat, Rev, NefI) [73,74], and also against outer membrane proteins of *Chlamydia trachomatis* [75]. Antibodies raised against the latter proteins detect the pathogen in sera of *Chlamydia*-positive patients. Additionally, the effector protein SopB of *Salmonella enterica* serovar *typhimurium* was fused to GvpC and the engineered gas vesicles injected into mice immunized with a live attenuated *Salmonella* vaccine strain [76]. The bacterial burden in mice boosted with SopB gas vesicles was reduced. In addition, the murine bactericidal permeability-increasing protein was displayed on gas vesicles and rescue mice from lethal endotoxic shock [77].

Another application is the use of gas vesicles derived from *Hbt. salinarum, Anabaena flos aquae*, or *Bacillus megaterium* as a novel contrast agent for imaging by ultrasound [78]. Gas vesicles scatter sound waves and thus produce ultrasound contrasts when injected in mice. The advantage is that they are much more stable compared to the inherently unstable synthetic micron bubbles usually applied for imaging [79]. They produce contrast in hyperpolarized xenon magnetic resonance imaging (MRI) and enable sensitive and noninvasive observations of the anatomy [80,81]. Conventional MRI contrast agents are based on super paramagnetic ion oxide or lathanide chelates that are potentially toxic and require high concentrations in the µM range for detection. In contrast, gas vesicles filled with hyperpolarized ^129^Xe are detected at picomolar concentrations through hyperpolarized chemical exchange saturation transfer (HyperCEST) pulse sequences [80,82].

Basic research on gas vesicles indicates that their shape and size affect the resistance to hydrostatic pressure, but gas vesicles also collapse at a critical acoustic pressure that is up to nine-fold higher than the critical hydrostatic pressure determined [83]. Gas vesicles of *Hbt. salinarum, A. flos aquae*, and *B. megaterium* show a differential response to hydrostatic or acoustic pressure. The halobacterial gas vesicles exhibit the largest diameters and are the weakest; they collapse with ultrasound at 6 MHz and a pressure of 98 kPa [83]. Apart from the genetic differences of bacterial or haloarchaeal gas vesicles, the mechanical and acoustic properties can be engineered by the replacement of GvpC on the surface by a modified recombinant GvpC protein [84]. His-tagged GvpC_WT_ or a smaller GvpC variant lacking the N- and C-terminal portions of *Anabaena* GvpC were produced in *Escherichia coli* and added to purified *Anabaena* gas vesicles stripped off the native GvpC with 6 M urea. The gas vesicles devoid of GvpC collapse under a lower acoustic pressure compared to the gas vesicles decorated with the GvpC deletion variant or with GvpC_WT_ [84]. Collapsing gas vesicles by ultrasound leads to the lack of their contrast and allows multiplexed imaging through serial collapse.

Gas vesicles are also engineered to allow an enhanced cell-specific targeting [81,84]. The altered GvpC display peptides such as arginyl glycyl aspartic acid (RGD) that efficiently bind integrins, and gas vesicles decorated with GvpC_RGD_ target an integrin-overexpressing human glioblastoma cell line in vivo. A reduced uptake of gas vesicles by macrophages is achieved by decorating them with GvpC_CD47_ harboring a peptide of the mammalian membrane protein CD47 at the C-terminus, and the presence of the peptide polyarginine R8 (GvpC_R8_) leads to a more efficient gas vesicles take-up. Such GvpC modifications allow cellular labeling by gas vesicles.

A more recent application is the use of gas vesicles as biosensors for ultrasound imaging of enzyme activities [85]. *Anabaena* GvpC proteins are engineered by incorporating a specific protease recognition sequence in the second helical repeat, such as the recognition sequences of the endopeptidase TEV of the tobacco etch virus or of the calcium-dependent mammalian protease calpin. The rationale is that the recognition motif in GvpC is recognized by the respective protease, and GvpC is cleaved by TEV or calpin into two smaller fragments. Even if the fragments are still attached, the resulting gas vesicles are less stiff and undergo buckling and produce an enhanced nonlinear ultrasound contrast. The critical hydrostatic pressure of TEV-treated GvpC_TEV_ gas vesicles is indeed reduced, and they produce a more nonlinear contrast, whereas gas vesicles decorated with GvpC_WT_ show no difference in their hydrostatic collapse pressure or in the nonlinear acoustic contrast after TEV treatment [85]. The insertion of the recognition sequence for the calcium-dependent protease calpin within *Anabaena* GvpC yields gas vesicles showing a 50-kPa decrease in the hydrostatic collapse pressure in calpin- plus Ca^++^-containing buffers compared to buffers without calpin and a robust nonlinear response by ultrasound imaging [85]. The latter biosensor can be used to visualize the dynamic response of calpin to Ca^++^ by ultrasound imaging.

Additionally tested was the bacterial ClpXP, an ATP-powered unfolding and protein degradation machine [86]. The specific terminal ClpXP degron was fused to the C-terminus of GvpC, and the sequence is recognized by the unfoldase ClpX, unfolded and fed into ClpP for degradation [85]. The addition of ClpXP leads to the degradation of GvpC but leaves the underlying GvpA shell of the gas vesicle intact. The resulting gas vesicles have an increased mechanical flexibility and exhibit a nonlinear ultrasound contrast. The system was tested in vivo in *E. coli* Nissle 1917 cells. GvpC_ClpXP_-gas vesicles produced under native *glpXP* expression in *E. coli* exhibit a significantly reduced collapse pressure and an enhanced nonlinear contrast in ultrasound imaging. The expression of ClpXP under the control of an arabinose-inducible promoter allows to control the ClpXP activity externally, and after induction with L-arabinose, the hydrostatic collapse pressure of the gas vesicles is strongly reduced (by 160 kPa), and a substantially stronger nonlinear contrast is observed when cells are imaged by ultrasound [85]. Such acoustic biosensors can be applied to measure gene expression, for example, in synthetic genetic circuits. In all these cases, GvpC variants allow the modulation of the mechanical, acoustic, surface, and targeting properties of gas vesicles. Future research will certainly advance the use of gas vesicles as acoustic reporters in mammalian cells [87].

## 8. Conclusions

Gas vesicles are unique intracellular structures used by bacteria and archaea to thrive in regions that are optimal for growth. They have been studied with respect to the expression and regulation of the *gvp* genes involved, their mechanical properties, their adaptation to environmental conditions, their assembly, and their evolutionary relationships over the past 50 years. The size, shape, and the strength of the gas vesicle shell is genetically determined, and the process of self-assembly of these nanostructures is not yet fully understood. However, interesting applications for gas vesicles have been developed over the past years. Gas vesicles scatter waves and have proved to be useful as acoustic biosensors. Their mechanical and biochemical properties can be modulated by altering the surface protein GvpC, and functionalized gas vesicles increase their use as acoustic reporters or as tool to present peptides of pathogens for the development of novel vaccines. Future studies will focus on the further development as biosensors but also on basic research to completely understand the steps driving the self-assembly process leading to the gas vesicle shell. Determination of the 3D structure of GvpA assembled in the shell by cryo-electron tomography and of the exact location of GvpC dimers or multimers at the gas vesicle surface are major questions to be solved in the future. In addition, localizing the different accessory Gvp proteins in the gas vesicle structure would be rewarding and also help to determine whether these proteins are permanently present or only found in juvenile gas vesicle structures.

## Figures and Tables

**Table 1:** Gas vesicle gene clusters of bacterial and archaeal species. (+) indicates that genes or gene clusters are arranged in opposite directions and (−) that *gvp* genes are separated by unrelated genes not involved in gas vesicle formation; (/) describes two consecutive operons. * *gvp* gene clusters identified in genome sequences deposited at the NCBI.

Species	*gvp* Gene Cluster(s)
*Halobacterium salinarum* *Haloferax mediterranei* *Halogeometricum borinquense* *	*gvpACNO + gvpDEFGHIJKLM*
*Haloquadratum walsbyi*	*gvpACNO/gvpFGHIJKLM*
*Halorubrum vacuolatum*	*gvpACNOFGHIJKLM*
*Natrialba magadii* * Halopiger xanaduensis * Haloadaptus paucihalophilus * Natrinema pellirubrum * Natronobacterium gregoryi *	*gvpACNO + gvpFGHIJKLM*
*Methanosarcina barkeri*	*gvpAAA–NOFG–JHKLM*
*Anabaena flos aquae*	*gvpAAAAAAACNJKFGVW*
*Microcystis aeruginosa*	*gvpAACNJXKFG + V + W*
*Bacillus megaterium*	*gvpAPQBRNFGLSKJTU*
*Serratia* sp. ATCC39006	*gvpA_1_CNVF_1_GWA_2_KXA_3_Y*/*gvrAgvpHZF_2_F_3_gvrBgvrC*
*Streptomyces coelicolor*	*gvpOAFG–JLSK*

**Table 2:** Functions of the different Gvp proteins derived from *Hbt. salinarum*. The accessory Gvp are present in minor amounts and are either essential or not essential for gas vesicle formation, as determined by ∆X transformants [31].

Protein	Size (kDa)	Putative Functions and Remarks
GvpA	8.0	Major gas vesicle structural protein, amphiphilic. Sequence similarity to GvpJ (50%) and GvpM (48%). Forms the helical ribs of the gas vesicle wall by aggregation. Structural model indicates a coil-α-β-β-α-coil structure.
GvpC	42.3	Gas vesicle structural protein, attached to the exterior surface and reinforcing the shell. Contains 6–7 aa repeats of α-helical structure, 32–40 aa in length near the N-terminus; the globular C-terminal domain contains a zinc-finger motif. Determines the cylindrical shape.
GvpF	24.0	Essential accessory Gvp; similarity to GvpL. The only Gvp protein interacting with GvpA in split-GFP analysis; interacts with other Gvp.
GvpG	10.0	Essential accessory Gvp; interacts with other Gvp.
GvpH	19.8	Non-essential accessory Gvp. Gas vesicles formed in ∆H transformants are weaker compared to wild type. Prevents GvpM aggregation; heterodimer formed with GvpI.
GvpI	15.8	Non-essential accessory Gvp; basic pI of 10.8. ∆I transformants contain longer gas vesicles than wild type. Interacts with other Gvp; heterodimer formed with GvpH.
GvpJ	11.9	Essential hydrophobic accessory Gvp; sequence similarity to GvpA (50%) and GvpM (60%). Homology modelling suggests a structure similar to GvpA. Interacts with other Gvp.
GvpK	12.6	Essential accessory Gvp. Member of the putative nucleation complex. Forms multimers in the presence of GvpI. Interacts with other Gvp proteins
GvpL	32.0	Essential accessory Gvp, structural homology to GvpF; Interacts with any other Gvp protein except for GvpA; platform for the nucleation complex.
GvpM	9.2	Essential hydrophobic accessory Gvp; similarity to GvpA (48%) and GvpJ (60%). Required for initial steps in the gas vesicle formation. Interacts with other Gvp.
GvpN	39.0	Contains a NTP binding/AAA+ domain; hydrolyzes ATP in cyanobacteria [32]. Required to enlarge the bicones into cylinder-shaped gas vesicles. Interacts with any Gvp.
GvpO	13.2	Essential accessory Gvp of unknown function. Interacts with GvpA and several other Gvp.
GvpD	59.3	Regulator protein with repressing function; NTP-binding domain is essential for its function; presence of GvpD leads to the degradation of GvpE [33,34,35].
GvpE	20.9	Transcriptional activator acting at the divergent promoters *P_pA_* and *P_pD_* of the p-vac region; the 20-nt GvpE-responsive element (UAS) is located upstream and adjacent to BRE/TATA-box of both promoters. Both UAS overlap in the center of the 35-nt intergenic region [36,37,38].

**Table 3:** Protein–protein interactions according to the rf values determined by split-GFP assays. The Gvp proteins investigated derive from *Hbt. salinarum* PHH1.

Gvp *	rf > 20	rf 10–20	rf 5–10	rf 1–5	rf < 1
GvpA	F	--	--	N,L	A,J,M,C,O,G,H-I,K
GvpC	L	C,I	N,F,H,K	O,G,J	A,M
GvpN	--	N-O,L	C,F,G,H-I,J,M,K	A	--
GvpO	--	N-O	C,F,I,L	G,H,J,K	A,M
GvpF	A	L	C,O,G,H-I	N,K,M	J
GvpG	L	--	F	C,N-O,I,K	A,J,M,H
GvpH	I	L	C,F	N-O	A,J,M,G,K
GvpI	H	C,L	O,F	N,G,K,J,M	A
GvpJ	--	L	--	C,N-O,I,K	A,M,F,G,H
GvpK	--	--	C,L	N-O,F,G,I,J,M	A,H
GvpL	C,G	N-O,F,H-I,J,M	K	A	--
GvpM	--	L	--	N-O,F,I,K	A,J,C,G,H

* Red, major interaction protein GvpL; blue, major interaction protein GvpC; yellow, H-I heterodimer; green, N-O heterodimer; grey, hydrophobic proteins of the A-J-M family. An rf-value < 5 is regarded as a very low interaction (after [50,58]).

**Table 4:** Interactions determined by a pulldown assay with CBD as the tag. GvpF through GvpM were not tested with GvpA, C, N, or O, and GvpC, N, and O were not tested with GvpF through GvpM. The Gvp proteins investigated derive from *Hbt. salinarum* PHH1.

Gvp	Interactions Observed [50,58] *
GvpA	F, G, H, J monomer + dimer, M monomer
GvpC	N, O multimer
GvpN	A multimer, C, O multimer
GvpO	A mono- + multimer, C, N
GvpF	G, H-I, J multimer, K, L, M
GvpG	F, H-I, J monomer, K, L, M
GvpH	F, G dimer, I, J multimer, K, L, M monomer
GvpI	F, G dimer, H, J multimer, K multimer, L, M
GvpJ	F, G, H-I, K, L, M
GvpK	F, G, H-I, J, L, M
GvpL	F, G dimer, J monomer, K, M monomer
GvpM	F, G, H-I, J mono- + multimer, K, L

* Red, major interaction protein GvpL; blue, major interaction protein GvpC; yellow, H-I heterodimer; grey, hydrophobic proteins of the A-J-M family.

## Data Availability

Not applicable.

## References

[1] Walsby A.E. (1969). The permeability of blue-green algal gas-vacuole membranes to gas. Proc. R. Soc. Lond B Biol. Sci..

[2] Walsby A.E. (1994). Gas vesicles. Microbiol. Rev..

[3] Thomas R.H., Walsby A.E. (1985). Buoyancy regulation in a strain of *Microcystis*. J. Gen. Microbiol..

[4] Oliver R.L., Walsby A.E. (1984). Direct evidence for the role of light-mediated gas vesicle collapse in the buoyancy regulation of *Anabaena flos-aquae* (Cyanobacteria). Limnol. Oceanogr..

[5] Walsby A.E., Mcallister G.K. (1987). Buoyancy regulation by *Microcystis* in Lake Okaro. N. Z. J. Mar. Fresh.

[6] Staley J.T., Irgens R.L., Brenner D.J. (1987). *Enhydrobacter aerosaccus* gen. nov., sp. nov, a gas-vacuolated, facultatively anaerobic, heterotrophic rod. Int. J. Sys. Bacteriol..

[7] Staley J.T., Irgens R.L., Herwig R.P. (1989). Gas vacuolate bacteria from the sea ice of Antarctica. Appl. Environ. Microbiol..

[8] Jung D.O., Achenbach L.A., Karr E.A., Takaichi S., Madigan M.T. (2004). A gas vesiculate planktonic strain of the purple non-sulfur bacterium *Rhodoferax antarcticu*s isolated from Lake Fryxell, Dry Valleys, Antarctica. Arch. Microbiol..

[9] Li N., Cannon M.C. (1998). Gas vesicle genes identified in *Bacillus megaterium* and functional expression in *Escherichia coli*. J. Bacteriol..

[10] van Keulen G., Hopwood D.A., Dijkhuizen L., Sawers R.G. (2005). Gas vesicles in actinomycetes: Old buoys in novel habitats?. Trends Microbiol..

[11] Ramsay J.P., Williamson N.R., Spring D.R., Salmond G.P. (2011). A quorum-sensing molecule acts as a morphogen controlling gas vesicle organelle biogenesis and adaptive flotation in an enterobacterium. Proc. Natl. Acad. Sci. USA.

[12] Ramsay J.P., Salmond G.P. (2012). Quorum sensing-controlled buoyancy through gas vesicles: Intracellular bacterial microcompartments for environmental adaptation. Commun. Integr. Biol..

[13] Stoeckenius W., Kunau W.H. (1968). Further characterization of particulate fractions from lysed cell envelopes of *Halobacterium halobium* and isolation of gas vacuole membranes. J. Cell Biol..

[14] Englert C., Wanner G., Pfeifer F. (1992). Functional analysis of the gas vesicle gene cluster of the halophilic archaeon *Haloferax mediterranei* defines the vac-region boundary and suggests a regulatory role for the *gvpD* gene or its product. Mol. Microbiol..

[15] Bolhuis H., te Poele E.M., Rodriguez-Valera F. (2004). Isolation and cultivation of Walsby’s square archaeon. Environ. Microbiol..

[16] Burns D.G., Camakaris H.M., Janssen P.H., Dyall-Smith M.L. (2004). Cultivation of Walsby’s square haloarchaeon. FEMS Microbiol. Lett..

[17] Englert C., Horne M., Pfeifer F. (1990). Expression of the major gas vesicle protein gene in the halophilic archaebacterium *Haloferax mediterrane*i is modulated by salt. Mol. Gen. Genet..

[18] Bleiholder A., Frommherz R., Teufel K., Pfeifer F. (2012). Expression of multiple *tfb* genes in different *Halobacterium salinarum* strains and interaction of TFB with transcriptional activator GvpE. Arch. Microbiol..

[19] Hechler T., Pfeifer F. (2009). Anaerobiosis inhibits gas vesicle formation in halophilic Archaea. Mol. Microbiol..

[20] Pfeifer F. (2015). Haloarchaea and the formation of gas vesicles. Life.

[21] Maeder D.L., Anderson I., Brettin T., Bruce D., Gilna P., Han C., Lapidus A., Metcalf W., Saunders E., Tapia R. (2006). The *Methanosarcina barkeri* genome: Comparative analysis with *Methanosarcina acetivorans* and *Methanosarcina mazei* reveals extensive rearrangement within methanosarcinal genomes. J. Bacteriol..

[22] Englert C., Krüger K., Offner S., Pfeifer F. (1992). Three different but related gene clusters encoding gas vesicles in halophilic archaea. J. Mol. Biol..

[23] Ng W.V., Kennedy S.P., Mahairas G.G., Berquist B., Pan M., Shukla H.D., Lasky S.R., Baliga N.S., Thorsson V., Sbrogna J. (2000). Genome sequence of *Halobacterium* species NRC-1. Proc. Natl. Acad. Sci. USA.

[24] Offner S., Wanner G., Pfeifer F. (1996). Functional studies of the *gvpACNO* operon of *Halobacterium salinarium* reveal that the GvpC protein shapes gas vesicles. J. Bacteriol..

[25] Pfeifer F. (2012). Distribution, formation and regulation of gas vesicles. Nat. Rev. Microbiol..

[26] Kinsman R., Hayes P.K. (1997). Genes encoding proteins homologous to halobacterial Gvps N, J, K, F & L are located downstream of *gvpC* in the cyanobacterium *Anabaena flos-aquae*. DNA Seq..

[27] Mlouka A., Comte K., Castets A.M., Bouchier C., Tandeau de Marsac N. (2004). The gas vesicle gene cluster from *Microcystis aeruginosa* and DNA rearrangements that lead to loss of cell buoyancy. J. Bacteriol..

[28] Tashiro Y., Monson R.E., Ramsay J.P., Salmond G.P. (2016). Molecular genetic and physical analysis of gas vesicles in buoyant enterobacteria. Environ. Microbiol..

[29] Tavlaridou S., Faist K., Weitzel K., Pfeifer F. (2013). Effect of an overproduction of accessory Gvp proteins on gas vesicle formation in *Haloferax volcanii*. Extremophiles.

[30] Monson R.E., Tashiro Y., Salmond G.P.C. (2016). Overproduction of individual gas vesicle proteins perturbs flotation, antibiotic production and cell division in the enterobacterium *Serratia* sp. ATCC 39006. Microbiology.

[31] Offner S., Hofacker A., Wanner G., Pfeifer F. (2000). Eight of fourteen *gvp* genes are sufficient for formation of gas vesicles in halophilic archaea. J. Bacteriol..

[32] Cai K., Xu B.Y., Jiang Y.L., Wang Y., Chen Y., Zhou C.Z., Li Q. (2020). The model cyanobacteria *Anabaena* sp. PCC 7120 possess an intact but partially degenerated gene cluster encoding gas vesicles. BMC Microbiol..

[33] Pfeifer F., Zotzel J., Kurenbach B., Röder R., Zimmermann P. (2001). A p-loop motif and two basic regions in the regulatory protein GvpD are important for the repression of gas vesicle formation in the archaeon *Haloferax mediterranei*. Microbiology.

[34] Zimmermann P., Pfeifer F. (2003). Regulation of the expression of gas vesicle genes in *Haloferax mediterranei*: Interaction of the two regulatory proteins GvpD and GvpE. Mol. Microbiol..

[35] Scheuch S., Pfeifer F. (2007). GvpD-induced breakdown of the transcriptional activator GvpE of halophilic archaea requires a functional p-loop and an arginine-rich region of GvpD. Microbiology.

[36] Plößer P., Pfeifer F. (2002). A bZIP protein from halophilic archaea: Structural features and dimer formation of cGvpE from *Halobacterium salinarum*. Mol. Microbiol..

[37] Gregor D., Pfeifer F. (2005). In vivo analyses of constitutive and regulated promoters in halophilic archaea. Microbiology.

[38] Marschaus L., Pfeifer F. (2012). A dual promoter region with overlapping activator sequences drives the expression of gas vesicle protein genes in haloarchaea. Microbiology.

[39] Offner S., Pfeifer F. (1995). Complementation studies with the gas vesicle-encoding p-vac region of *Halobacterium salinarium* PHH1 reveal a regulatory role for the p-*gvpDE* genes. Mol. Microbiol..

[40] Born J., Pfeifer F. (2019). Improved GFP variants to study gene expression in haloarchaea. Front. Microbiol..

[41] Holmes M.L., Scopes R.K., Moritz R.L., Simpson R.J., Englert C., Pfeifer F., Dyall-Smith M.L. (1997). Purification and analysis of an extremely halophilic beta-galactosidase from *Haloferax alicantei*. Biochim. Biophys. Acta.

[42] Bauer M., Marschaus L., Reuff M., Besche V., Sartorius-Neef S., Pfeifer F. (2008). Overlapping activator sequences determined for two oppositely oriented promoters in halophilic archaea. Nucleic Acids Res..

[43] Hayes P.K., Walsby A.E., Walker J.E. (1986). Complete amino acid sequence of cyanobacterial gas vesicle protein indicates a 70-residue molecule that corresponds in size to the crystallographic unit cell. Biochem. J..

[44] Hayes P.K., Buchholz B., Walsby A.E. (1992). Gas vesicles are strengthened by the outer-surface protein, GvpC. Arch. Microbiol..

[45] Walsby A.E., Hayes P.K. (1988). The minor cyanobacterial gas vesicle protein, GvpC, is attached to the outer surface of the gas vesicle. J. Gen. Microbiol..

[46] Englert C., Pfeifer F. (1993). Analysis of gas vesicle gene expression in *Haloferax mediterranei* reveals that GvpA and GvpC are both gas vesicle structural proteins. J. Biol. Chem..

[47] Halladay J.T., Jones J.G., Lin F., MacDonald A.B., DasSarma S. (1993). The rightward gas vesicle operon in *Halobacterium* plasmid pNRC100: Identification of the *gvpA* and *gvpC* gene products by use of antibody probes and genetic analysis of the region downstream of *gvpC*. J. Bacteriol..

[48] Kinsman R., Walsby A.E., Hayes P.K. (1995). GvpCs with reduced numbers of repeating sequence elements bind to and strengthen cyanobacterial gas vesicles. Mol. Microbiol..

[49] Hechler T., Frech M., Pfeifer F. (2008). Glucose inhibits the formation of gas vesicles in *Haloferax volcanii* transformants. Environ. Microbiol..

[50] Völkner K., Jost A., Pfeifer F. (2020). Accessory *gvp* proteins form a complex during gas vesicle formation of haloarchaea. Front. Microbiol..

[51] Pfeifer F., Offner S., Krüger K., Ghahraman P., Englert C. (1994). Transformation of halophilic archaea and investigation of gas vesicle synthesis. Syst. Appl. Microbiol..

[52] Winter K., Born J., Pfeifer F. (2018). Interaction of haloarchaeal gas vesicle proteins determined by split-GFP. Front. Microbiol..

[53] Ghosh I., Hamilton A.D., Regan L. (2000). Antiparallel leucine zipper-directed protein reassembly: Application to the green fluorescent protein. J. Am. Chem. Soc..

[54] Magliery T.J., Wilson C.G.M., Pan W.L., Mishler D., Ghosh I., Hamilton A.D., Regan L. (2005). Detecting protein-protein interactions with a green fluorescent protein fragment reassembly trap: Scope and mechanism. J. Am. Chem. Soc..

[55] Finnigan G.C., Duvalyan A., Liao E.N., Sargsyan A., Thorner J. (2016). Detection of protein-protein interactions at the septin collar in *Saccharomyces cerevisiae* using a tripartite split-GFP system. Mol. Biol. Cell.

[56] Reuter C.J., Maupin-Furlow J.A. (2004). Analysis of proteasome-dependent proteolysis in *Haloferax volcanii* cells, using short-lived green fluorescent proteins. Appl. Environ. Microbiol..

[57] Tavlaridou S., Winter K., Pfeifer F. (2014). The accessory gas vesicle protein GvpM of haloarchaea and its interaction partners during gas vesicle formation. Extremophiles.

[58] Jost A., Pfeifer F. (2022). Interaction of the gas vesicle proteins GvpA, GvpC, GvpN, and GvpO of *Halobacterium salinarum*. Front. Microbiol..

[59] Strunk T., Hamacher K., Hoffgaard F., Engelhardt H., Zillig M.D., Faist K., Wenzel W., Pfeifer F. (2011). Structural model of the gas vesicle protein GvpA and analysis of GvpA mutants *in vivo*. Mol. Microbiol..

[60] Ezzeldin H.M., Klauda J.B., Solares S.D. (2012). Modeling of the major gas vesicle protein, GvpA: From protein sequence to vesicle wall structure. J. Struct. Biol..

[61] Jost A., Knitsch R., Volkner K., Pfeifer F. (2021). Effect of mutations in GvpJ and GvpM on gas vesicle formation of *Halobacterium salinarum*. Front. Microbiol..

[62] Knitsch R., Schneefeld M., Weitzel K., Pfeifer F. (2017). Mutations in the major gas vesicle protein GvpA and impacts on gas vesicle formation in *Haloferax volcanii*. Mol. Microbiol..

[63] Beard S.J., Hayes P.K., Pfeifer F., Walsby A.E. (2002). The sequence of the major gas vesicle protein, GvpA, influences the width and strength of halobacterial gas vesicles. FEMS Microbiol. Lett..

[64] Hayes P.K., Lazarus C.M., Bees A., Walker J.E., Walsby A.E. (1988). The protein encoded by *gvpC* is a minor component of gas vesicles isolated from the cyanobacteria *Anabaena flos-aquae* and *Microcystis* sp.. Mol. Microbiol..

[65] Buchholz B.E., Hayes P.K., Walsby A.E. (1993). The distribution of the outer gas vesicle protein, GvpC, on the *Anabaena* gas vesicle, and its ratio to GvpA. J. Gen. Microbiol..

[66] Dunton P.G., Walsby A.E. (2005). The diameter and critical collapse pressure of gas vesicles in *Microcystis* are correlated with GvpCs of different length. FEMS Microbiol. Lett..

[67] Dunton P.G., Mawby W.J., Shaw V.A., Walsby A.E. (2006). Analysis of tryptic digests indicates regions of GvpC that bind to gas vesicles of *Anabaena flos-aquae*. Microbiology.

[68] Xu B.Y., Dai Y.N., Zhou K., Liu Y.T., Sun Q., Ren Y.M., Chen Y., Zhou C.Z. (2014). Structure of the gas vesicle protein GvpF from the cyanobacterium *Microcystis aeruginosa*. Acta Crystallogr. D Biol. Crystallogr..

[69] Sivertsen A.C., Bayro M.J., Belenky M., Griffin R.G., Herzfeld J. (2010). Solid-state NMR characterization of gas vesicle structure. Biophys. J..

[70] Bayro M.J., Daviso E., Belenky M., Griffin R.G., Herzfeld J. (2012). An amyloid organelle, solid-state NMR evidence for cross-beta assembly of gas vesicles. J. Biol. Chem..

[71] DasSarma S., DasSarma P. (2015). Gas vesicle nanoparticles for antigen display. Vaccines.

[72] Adamiak N., Krawczyk K.T., Locht C., Kowalewicz-Kulbat M. (2021). Archaeosomes and gas vesicles as tools for vaccine development. Front. Immunol..

[73] Sremac M., Stuart E.S. (2008). Recombinant gas vesicles from *Halobacterium* sp. Displaying SIV peptides demonstrate biotechnology potential as a pathogen peptide delivery vehicle. BMC Biotechnol..

[74] Sremac M., Stuart E.S. (2010). SIVsm Tat, Rev, and Nef1: Functional characteristics of r-GV internalization on isotypes, cytokines, and intracellular degradation. BMC Biotechnol..

[75] Childs T.S., Webley W.C. (2012). In vitro assessment of halobacterial gas vesicles as a Chlamydia vaccine display and delivery system. Vaccine.

[76] DasSarma P., Negi V.D., Balakrishnan A., Karan R., Barnes S., Ekulona F., Chakravortty D., DasSarma S. (2014). Haloarchaeal gas vesicle nanoparticles displaying *Salmonella* SopB antigen reduce bacterial burden when administered with live attenuated bacteria. Vaccine.

[77] Balakrishnan A., DasSarma P., Bhattacharjee O., Kim J.M., DasSarma S., Chakravortty D. (2016). Halobacterial nano vesicles displaying murine bactericidal permeability-increasing protein rescue mice from lethal endotoxic shock. Sci. Rep..

[78] Shapiro M.G., Goodwill P.W., Neogy A., Yin M., Foster F.S., Schaffer D.V., Conolly S.M. (2014). Biogenic gas nanostructures as ultrasonic molecular reporters. Nat. Nanotechnol..

[79] Mukherjee A., Davis H.C., Ramesh P., Lu G.J., Shapiro M.G. (2017). Biomolecular MRI reporters: Evolution of new mechanisms. Prog. Nucl. Magn. Reson. Spectrosc..

[80] Shapiro M.G., Ramirez R.M., Sperling L.J., Sun G., Sun J., Pines A., Schaffer D.V., Bajaj V.S. (2014). Genetically encoded reporters for hyperpolarized xenon magnetic resonance imaging. Nat. Chem..

[81] Lakshmanan A., Lu G.J., Farhadi A., Nety S.P., Kunth M., Lee-Gosselin A., Maresca D., Bourdeau R.W., Yin M., Yan J. (2017). Preparation of biogenic gas vesicle nanostructures for use as contrast agents for ultrasound and MRI. Nat. Protoc..

[82] Farhadi A., Ho G., Kunth M., Ling B., Lakshmanan A., Lu G.J., Bourdeau R.W., Schroder L., Shapiro M.G. (2018). Recombinantly expressed gas vesicles as nanoscale contrast agents for ultrasound and hyperpolarized MRI. Aiche J..

[83] Cherin E., Melis J.M., Bourdeau R.W., Yin M., Kochmann D.M., Foster F.S., Shapiro M.G. (2017). Acoustic behavior of *Halobacterium salinarum* gas vesicles in the high-frequency range: Experiments and modeling. Ultrasound Med. Biol..

[84] Lakshmanan A., Farhadi A., Nety S.P., Lee-Gosselin A., Bourdeau R.W., Maresca D., Shapiro M.G. (2016). Molecular engineering of acoustic protein nanostructures. Acs Nano.

[85] Lakshmanan A., Jin Z., Nety S.P., Sawyer D.P., Lee-Gosselin A., Malounda D., Swift M.B., Maresca D., Shapiro M.G. (2020). Acoustic biosensors for ultrasound imaging of enzyme activity. Nat. Chem. Biol..

[86] Baker T.A., Sauter R.T. (2012). ClyXP, an ATP-powered unfolding and protein-degradation machine. Biochim. Biophys. Acta.

[87] Maresca D., Lakshmanan A., Abedi M., Bar-Zion A., Farhadi A., Lu G.J., Szablowski J.O., Wu D., Yoo S., Shapiro M.G. (2018). Biomolecular ultrasound and sonogenetics. Annu. Rev. Chem. Biomol. Eng..

